# Application of Spectroscopic UV-Vis and FT-IR Screening Techniques Coupled with Multivariate Statistical Analysis for Red Wine Authentication: Varietal and Vintage Year Discrimination

**DOI:** 10.3390/molecules24224166

**Published:** 2019-11-17

**Authors:** Elisabeta-Irina Geană, Corina Teodora Ciucure, Constantin Apetrei, Victoria Artem

**Affiliations:** 1 National R&D Institute for Cryogenics and Isotopic Technologies—ICIT Rm. Valcea, 4th Uzinei Street, PO Raureni, Box 7, 240050 Rm. Valcea, Romania; irina.geana@icsi.ro (E.-I.G.); corina.ciucure@icsi.ro (C.T.C.); 2 Physics and Environment, Department of Chemistry, Faculty of Science and Environment, “Dunarea de Jos” University of Galati, 111 Domneasca Street, RO-800008 Galati, Romania; 3 Research Station for Viticulture and Oenology Murfatlar, Calea Bucuresti str., no. 2, Murfatlar, 905100 Constanta, Romania; artemvictoria@yahoo.com

**Keywords:** wine authentication, chemometrics, spectroscopic techniques, UV-Vis, FT-IR

## Abstract

One of the most important issues in the wine sector and prevention of adulterations of wines are discrimination of grape varieties, geographical origin of wine, and year of vintage. In this experimental research study, UV-Vis and FT-IR spectroscopic screening analytical approaches together with chemometric pattern recognition techniques were applied and compared in addressing two wine authentication problems: discrimination of (i) varietal and (ii) year of vintage of red wines produced in the same oenological region. UV-Vis and FT-IR spectra of red wines were registered for all the samples and the principal features related to chemical composition of the samples were identified. Furthermore, for the discrimination and classification of red wines a multivariate data analysis was developed. Spectral UV-Vis and FT-IR data were reduced to a small number of principal components (PCs) using principal component analysis (PCA) and then partial least squares discriminant analysis (PLS-DA) and linear discriminant analysis (LDA) were performed in order to develop qualitative classification and regression models. The first three PCs used to build the models explained 89% of the total variance in the case of UV-Vis data and 98% of the total variance for FR-IR data. PLS-DA results show that acceptable linear regression fits were observed for the varietal classification of wines based on FT-IR data. According to the obtained LDA classification rates, it can be affirmed that UV-Vis spectroscopy works better than FT-IR spectroscopy for the discrimination of red wines according to the grape variety, while classification of wines according to year of vintage was better for the LDA based FT-IR data model. A clear discrimination of aged wines (over six years) was observed. The proposed methodologies can be used as accessible tools for the wine identity assurance without the need for costly and laborious chemical analysis, which makes them more accessible to many laboratories.

## 1. Introduction

The consumers have been increasingly interested in information on the characteristics and the quality of wine, especially with regard to composition, nutritional properties, and origin, and for that, establishing its authenticity is one of the most important aspects in food quality and safety. Wine is one of the most susceptible products to adulteration, despite that there are specific and strict regulations protecting its authenticity [1]. Determination of wine authenticity by analytical methods has the purpose of confirming label declarations and is of great interest to the industry and consumers. As the adulteration of wine is an ongoing problem, there is a need of suitable analytical approaches to get more insight into the chemical composition and its changes associated with adulteration [2].

In order to keep up wine reputation and to minimize malpractices (mainly sugaring and watering), an official method was adopted by the European Union (EU) and by the International Organization of Vine and Wine (OIV) that requires reporting the isotopic pattern of the sample to the reference data base with isotopic parameters for authentic wine samples collected from across the EU [3].

One of the possible and common adulterations of wine, besides sugaring and the watering, is the falsification of the geographical and varietal origins and vintage year. In this respect, major wine-producing countries have developed severe Appellation Control laws that regulate the use of regional names for wines, providing the reference for delimiting the geographical origin of the wines, which is considered a fundamental indication of quality for consumers [4]. Wine marketing strategies focus on associating the product image and the perception of quality with a specific region and/or variety, increasing the importance of regional and varietal characteristics [5].

Wine authentication in terms of geographical and varietal origins, vintage year, identifying fraud, and determining the specifications of the product with the label are requirements of consumers and the European Community. For optimal solving of this problem, development and harmonization of validated analytical methods at a national and European level is necessary to improve the efficiency of wine control, this being one of the international priorities. One of the objectives of the European Reference Centre for Control in the Wine Sector (ERC-CWS) is to highlight the most promising analytical methods used for the discrimination of geographical origin, varietal composition, and vintage of wines [6]. However, it is necessary to identify the best strategy, the most promising analytical method(s) considering economic aspects (screening vs. confirmatory methods) to address this topic. In this context, spectroscopic techniques have become one of the most attractive and common methods of analysis providing rapid and accurate results [7].

In order to prove the wine quality warranty for the consumer, numerous researchers studied the authenticity of a wine using labor-intensive and costly analyses which look for specific chemical features, such as elemental profile [8], isotopic fingerprints [9], and organic compounds (i.e., phenolic and volatile compounds, amino acids, sugars) [10,11,12,13] that can be related to geographical origin, varietal composition, or vintage year. 

The composition of wine is influenced by many factors related to the specific production area: grape varieties, soil, weather, crop, and wine making practices [14]. Although the wine minoritary components (phenolic and volatile compounds, amino acids, organic acids, vitamins) may be influenced by the vinification, maturation, and aging, the differences in the overall phenolic fingerprints might still be characteristic for each cultivar [15].

For the varietal origin classification of wines, chromatography hyphenated to mass spectrometry [16,17] or other detectors as well as proton Nuclear magnetic resonance (^1^H-NMR) [18,19] and DNA-based methods seem to be most appropriate [20]. Nonetheless, the possibility to add legally up to 15% of another wine without any declaration is complicating the analysis. With the advent of metabolomics, it has been shown that ^1^H-NMR is a general tool in complex mixture analysis and also offers unique screening capabilities for food quality and safety by combining non-targeted and targeted screening in one measurement [19]. Conditions to implement this method in the official control laboratories are under discussion.

Most wines are consumed after a period of aging time in which important and diverse physicochemical changes occur during this period, and thus the aging process of the wine is associated with a high financial cost that must be recovered in the final price of the wine [10]. Therefore, the wine industry needs analytical tools to verify the authenticity of high quality wines like aged wines. 

Developing new alternative, accessible, and cost-effective innovative methods for wine authenticity assessment is something that attracts the attention of the industrial sectors representing a necessity at national and international level. Switching from target methods to non-targeted methods for food fraud testing, as in the case of screening spectroscopic techniques [21] including FT-IR [22,23], UV-Vis [24,25], NIR [26], and Raman [27] combined with multivariate statistical data analysis (MVA), has gained a wide acceptance for wine classification being easier, rapid, non-destructive, and providing useful information with good classification rates. Moreover, electronic noses and tongues are frequently used in the wine industry to evaluate the quality of wines, monitoring the aging of wines, or to discriminate between different wine categories [28]. However, the potential of screening techniques in wine control is strongly dependent on the set of authentic samples and the statistical approaches that are used to create the classification model [24]. 

There seems to be a problem, however, with treating a large quantity of data and for that, multivariate statistical data analysis (MVA), called chemometrics, is therefore required to extract the information about quality attributes which is buried by screening techniques. Developments in MVA, such as principal components analysis (PCA), linear discriminant linear analysis (LDA), soft independent modeling of class analogy (SIMCA), partial least squares (PLS), regression and qualitative techniques as cluster analysis (CA) in combination with different pre-processing methods, are applied to extract the required information from the convoluted spectra [29].

There is a real need for controlling the wine’s geographical and varietal origins and vintage year, and Romania made some contributions in this direction. The most published papers refer to the geographic origin authentication of wines [30,31,32,33], while the varietal and vintage classification of wines was performed for a small number of Romanian wine samples using analytical techniques like HPLC [34,35], HPTLC [36], quantitative UV-Vis spectrophotometry [37,38], and spectroscopic techniques like FT-MIR [39,40] and NMR [41].

The aim of this research was to investigate the applicability of UV-Vis and FT-IR screening spectroscopic techniques combined with multivariate statistical tools to classify red wines according to varietal origin and vintage year. As a case study, it were studied different red wine varieties produced at SCDVV Murfatlar (Dobrogea region, Romania) during nine years of production (from 2009 to 2017). The reason of this study was to identify the most promising methodology (spectroscopic technique combined with statistical method) for the discrimination of the wines according to the variety and vintage year, as an alternative, rapid, simple, and economical approach for wine authentication.

## 2. Results and Discussion

### 2.1. Spectral Characterization

#### 2.1.1. UV-Vis Absorption Spectra Analysis

The raw UV-Vis absorption spectra of red wines with different aging periods are visually very similar. The UV-Vis electromagnetic spectrum does indeed contain distinguishing features for wines (Supplementary Figure S1A,B). 

From the UV-Vis absorption spectra, the 250–600 nm region is of interest, being associated with volatile compounds and polyphenols originating from the grapes and the subsequent fermentation and aging process, which contain π conjugated systems with hydroxyl-phenolic groups. A strong absorption band around 280/290 nm was observed and which was associated with colorless compounds (flavanol monomers, flavanol polymers or tannins, etc.). The absorption intensity around 520 nm in the visible region of the electromagnetic spectrum is characteristic to the red coloring substances (anthocyanin compounds). The shoulders from 310 nm and 320 nm are characteristic to the galloyl group (galloylated flavanols) and acylated anthocyanins (malvidin-3-p-coumarylglucoside), respectively [42]. 

By comparing all of the spectra, noticeable differences in the spectra were found among the different red wine varieties and between wines belonging to the same varieties but with different harvest years. The peak values of absorbance varied with the variety and aging period. In concordance with [24], slight peak absorption wavelength shifts were observed among the different spectra of the wines with different aging periods.

#### 2.1.2. FT-IR Spectra Analysis

The Fourier Transform Infrared (FT-IR) spectra of different red wine varieties and different harvest years are visually very similar (Supplementary Figure S2). The spectra of all wines showed similar peaks. Only minor differences can be observed in specific areas of the spectra. 

It was observed that water and ethanol absorption peaks dominate the spectrum. The broad peak found in the 4000–3000 cm^−1^ region is mainly due to the stretching vibration of the O–H bond of water, alcohols, and phenols. Other water-related absorption bands were found at around 950 and 1460 nm, which are related to the third overtone of O–H. Absorption peaks associated with alcohols were observed at 2850–2960 cm^−1^ related to a CH stretch, at 2200–2300 cm^−1^ related to C–H combination vibrations and overtones of ethanol and sugars, and at 1087 and 1050 cm^−1^ related to a CO stretch for primary alcohols and glycerol [43].

The peaks from the 3000–2800 cm^−1^ region are most likely due to the stretching vibration of bonds from multiple constituents of the wines with vibrations including C–H stretching of hydrocarbons, O–H stretching of carboxylic acids, and asymmetric stretching vibrations of C–H bonds of methyl groups (–CH3): polyols (glycerol), free phenolic acids, and catechins. The region between 2200–1000 cm^−1^ can be correlated with the C−OH stretching, CH3 bending, CH2 bending, C=C stretching, and C≡N stretching of the groups in compounds, such as phenols, alcohols, aldehydes, higher alcohols, polyols, acids, sugars, volatile acids, and amino acids. The region of absorptions from 1900–1600 cm^−1^ is related to O–H combinations, C–H3 stretch first overtone and C–H2, C–H stretch first overtones of ethanol, water, and glucose [43].

Our interest was focused on the 1600–900 cm^−1^ spectral region because in this area characteristic groups absorb and the ‘fingerprint’ region is included. Consequently, in this region any differences between the spectra can be detected. Generally, in the 1600–900 cm^−1^ region bands originating from wine phenols can be found. The region between 1450 and 1278 cm^−1^ is very complex and contains information relating to C=O stretching, C=C, CH2, and C–H for aldehydes, carboxylic acids, proteins, and esters, around 1457–1427 cm^−1^ [15]. The bands at 1200, 1110–1107, and 1068–1062 cm^−1^ correspond to stretching vibrations of C–O and O–H stretch second overtones from sugars and organic acids [15,43]. A detailed description of the most representative regions of the FT-IR spectrum is presented in Table 1.

### 2.2. Multivariate Statistical Analysis

In order to objectively study if these minor visual differences are related to wine variety and the aging process, a comparative chemometric study was carried out by using UV-Vis and FT-IR spectra data. 

In order to avoid strong absorption of water and spectral features that are not strictly related to wine composition such as ethanol, the 4000–3000 cm^−1^ and 1900–1600 cm^−1^ spectral regions were excluded from the FT-IR data prior to performing multivariate statistical analysis. Thus, mainly the “fingerprint” region of the FT-IR spectrum was selected for further statistical analysis since absorptions in this region are due mainly to the bending and skeletal vibrations associated with phenolic compounds [22].

Full UV-Vis and fragmented FT-IR absorption spectra of all 39 wine samples were subjected to chemometric analysis, without prior signal pretreatments. As a result, the data matrix is arranged in 156 rows (including replicates) and 610 columns (variables) in the case of UV-Vis data and 156 rows (including replicates) and 1055 columns in the case of FT-IR data.

#### 2.2.1. Principal Component Analysis (PCA)

In order to handle the high dimensionality and complex nature of collected UV-Vis and FT-IR spectral data, a preliminary stage of feature extraction was considered in order to compress the relevant information for our process. PCA allows the visualization of the information in the data set in a few principal components while retaining the maximum possible variability within that set. Principal component analysis (PCA) was used to reduce the dimensionality of the spectral data to a smaller number of components, facilitating the subsequent analysis and reducing the risk of incorrect inferences. PCA was performed on the UV-Vis and FT-IR spectra of the wine samples, separately, to examine the possible grouping of samples related to wine varieties and harvest years. The differences in the proportions and the compositions of the families of the natural wine components make the discrimination between different wine varieties and harvest years possible.

**Figure 1:**
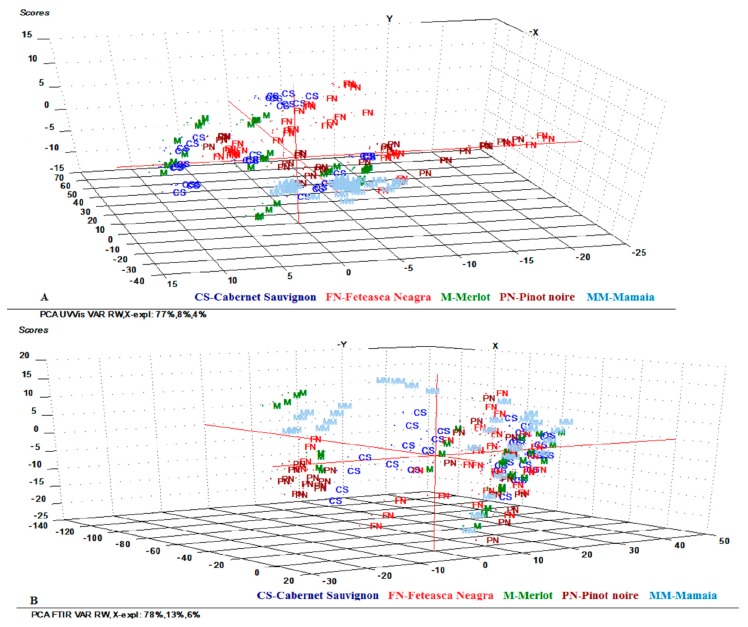
Score plot of the first 3 principal components (PCs) derived from (**A**) UV-Vis spectra of different red wine varieties and (**B**) FT–IR spectra of different red wine varieties.

Figure 1 shows the score plots of the UV-Vis and FT-IR data on the first three principal components (PCs), explaining 89% of the total variance of the UV-Vis data and 98% of the total variance of the FT-IR data.

For both, UV-Vis and FT-IR data, it was observed that the investigated red wine varieties overlapped in all plots, and thus incomplete separations between red wine varieties were observed. However, the best separation among red wine varieties was achieved using the FT-IR spectral data. In accordance with similar results reported by other authors, the PCA plot shows that the replicate samples are grouped in the same cluster, but without overlapping, which is due to bottle to bottle variation [46]. For each wine variety, it is worth noting that a dispersion of plots on the first three PCs dimension spaces was observed, being associated with the different climate of the harvest years investigated in this study. In addition, the trend of separation of Mamaia variety from the other red wine varieties was evident on the plot, which demonstrated the possibility of using PCA to distinguish this variety. 

In the case of harvest year discrimination, the scores for each sample on the first three PCs contain 89% of the total variance in the case of UV-Vis data and 97% of the total variance in the case of FT-IR data (Supplementary Figure S3). From the scatter plots, it could be discovered that wines of different years distributed separately in the three-dimension area. From the visual inspection of the PCA score plot of the investigated red wines, considering both UV-Vis and FT-IR spectral data, it was possible to discriminate the aged wines from the 2009, 2010, and 2011 harvest years from the rest of the other wines. As can be observed, wines aged for longer periods of time (more than six years: 2009, 2010, and 2011) showed the highest PC1 values, all of which fall in the positive area of PC1. On the contrary, the youngest samples (from 2012 to 2017), which showed the lowest PC1 values, fall into the negative area of PC1.

**Figure 2:**
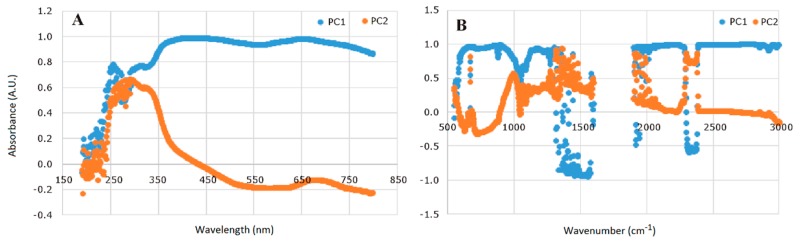
Eigenvector plot for the first 2 PCs: (**A**) UV-Vis data and (**B**) FT-IR data.

The eigenvectors of the first two PCs derived from the UV-Vis and FT-IR data were investigated to interpret the basis of the separation among wine varieties and harvest years. The loading values for the first two PCs obtained using UV-Vis and FT-IR data are represented in Figure 2. In the case of UV-Vis data, the greatest loading values (above 0.80) for the PC1 (Figure 2A) were observed at the wavelengths higher than 350 nm. This means that practically the whole visible range is affected by the differences between the varieties and aging process, the compounds responsible for this effect being the anthocyanin compounds. From the interpretation of the eigenvectors (loading values higher than 0.70) (Figure 2B), it was concluded that differences between the FT-IR spectra of red wines can be observed in the following regions: 600–900 cm^−1^ associated with phenolics and phenyl derivatives; 1100–1400 cm^−1^ associated with primary alcohols, glycerol, sugars (glucose and fructose), aromatic groups of phenolic compounds organic acids, and aldehydes, tannins, pigmented polymers [47]; 2000–2300 cm^−1^ related to alcohols, sugars, as well as compounds containing aromatic rings and organic acids [44].

#### 2.2.2. Partial Least Squares Discriminant Analysis (PLS-DA)

In wine analysis, the multivariate regression methods have been widely used to build calibration and prediction models, Partial Least Squares (PLS) regression being successfully applied for the determination of anthocyanins [48], antioxidant activity, total phenolic, and flavonoid contents [49].

In this study, PLS-DA models were developed using the spectral range selected previously by applying the PCA analysis, a number of 15 PCs being used to find PLS-DA models that allow the maximum separation among classes of different wine categories. The accuracy of PLS-DA models was evaluated by the slope of the regression line (R^2^) and the intercept of the regression line with the vertical axis (RMSEC—Root Mean Square Error of Calibration, and RMSEV—Root Mean Square Error of Validation). A value of R^2^ close to 1 indicates a linear relationship between the predicted and actual wine category. RMSEC refers to the uncertainty of calibration while RMSEV estimates how well the method will predict wine categories for unknown samples [22]. When the slope of the regression line was greater and the intercept was smaller, the predictive ability of the model was better [50].

Table 2 shows the results derived from the different considered samples datasets, resulting in different classification models: a model considering the wine varietal discrimination and a model considering the discrimination of wines by harvest year. As can be observed, higher values of the R^2^ and smaller values of RMSEC and RMSEV were obtained considering FT-IR spectral data, compared with the UV-Vis spectral data, indicating a good prediction capability of FT-IR based regression models, for both, varietal and harvest year discrimination. 

The regression models developed have proven to be good enough to correlate the classification criteria of studied wine with the FT-IR spectral data, the correlation coefficient (R^2^) ranging from 0.813–0.860 for wine varietal classification, and from 0.626–0.872 for vintage year classification. The calculated RMSEV values for the models ranged between 0.197–0.261 for UV-Vis data and 0.135–0.182 for FT-IR data in the case of wine varietal discrimination, while the RMSEV values for the models ranged between 0.174–0.243 for UV-Vis data and 0.108–0.184 for FT-IR data in the case of wine vintage year discrimination, indicating lower uncertainty values concerning the methods’ prediction ability when considering FT-IR data, for both, varietal and vintage year discrimination. These results suggest that wine variety and vintage year can be better estimated using FT-IR data, compared to UV-Vis data.

Good values of correlation coefficient (R^2^) were obtained for Merlot, Mamaia, and Pinot noire varieties (0.911, 0.918, and 0.918 respectively), with RMSEC values of 0.121, 0.115, and 0.104 and RMSEV values of 0.157, 0.151, and 0.135, which represents satisfactory statistical significant values. Wines from 2010 and 2015 vintage years show good correlation coefficients (R^2^) (0.922 and 0.900) and a lower RMSEC (0.085 and 0.114) and RMSEV (0.108 and 0.142).

#### 2.2.3. Linear Discriminant Analysis (LDA)

LDA was applied as a supervised method in order to classify the wines according to the grape variety and harvest year. Seeing as in all four data sets the number of variables were very high compared to the number of samples, LDA was always applied working on the scores of the first principal components: (1) 5PCs; (2) 10PCs; (3) 15PCs. LDA classification matrix for the cross-validation results of red wine varieties using (1) 3PCs; (2) 5PCs; (3) 10PCs were presented in Supplementary Table S1. For each data set, the number of principal components corresponding to higher total variance was always retained. It was observed that all first 15 PCs were required to adequately discriminate among varieties, corresponding to about 64.86% total variance for the UV-Vis data, while 43.59% total variance correspond to FT-IR data. Using a cross-validation technique to the UV-Vis spectroscopic data, higher prediction abilities were for Mamaia (86.96%) and Feteasca Neagra (73.08%) wines and a lower value for Cabernet Sauvignon (42.86%) and Pinot noire (52.94%) wines. In the case of FT-IR fingerprinting technique, the results of the cross-validation technique are less favorable, with prediction abilities ranging from 24.55% in the case of Merlot wines and 61.11% in the case of Pinot noire wines. 

**Figure 3:**
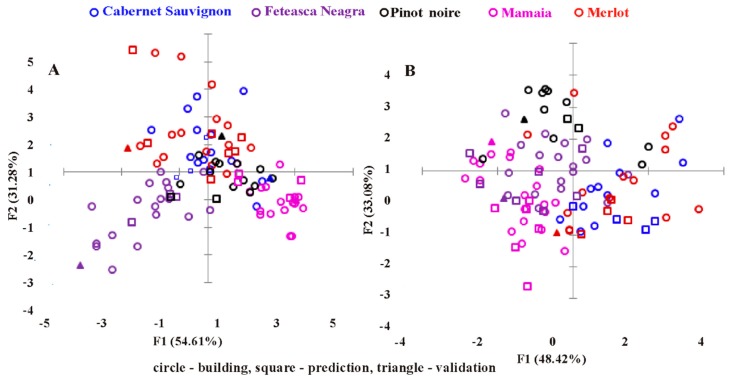
Scatter plot of the first two discriminant functions showing separation between wine varieties: (**A**) UV-Vis data and (**B**) FT-IR data.

The histograms on the LDA canonical variable for the UV-Vis and FT-IR data sets showing separation between wine varieties are presented in Figure 3.

Linear correlation revealed acceptable scores for two defined discriminant factors (F1 and F2). Using cross-validation technique, the results provided a percentage of predicted membership according to the wine variety of 85.89% (54.61% F1 and 31.28% F2) using UV-Vis data and 81.50% (48.42% F1 and 33.08% F2) using FT-IR data. 

As presented in Figure 3A, 85.89% of the samples were correctly classified using UV-Vis data, including the control wine samples, with a clear separation of Mamaia and Feteasca Neagra wines and an overlap for Pinot noire, Merlot, and Cabernet Sauvignon wines. The first discriminant function (F1) separated mainly Feteasca Neagra and Mamaia varieties, while the second one (F2) contributed to the discrimination of Feteasca Neagra wines from Cabernet Sauvignon and Merlot wines.

When FT-IR data were considered (Figure 3B), no clear separation between wines according to their varietal origin was shown, and the LDA score plots presented a considerable overlapping of the wines. The classification results (81.50% of the samples correctly classified) indicated that Mamaia, Merlot, and Cabernet Sauvignon wines can be associated with the first discriminant function (F1), while Pinot noire and Feteasca Neagra wines can be associated more with the second discrimination function (F2).

The comparison of the LDA results obtained from the UV-Vis and FT-IR fingerprinting techniques showed that the UV-Vis spectroscopic techniques worked better than FT-IR for the discrimination of wines according to the grape variety. The better classification using UV-Vis data compared to FT-IR data suggests that the differences among different wine varieties can be attributed to the colored phenolic compounds that absorb in the UV-Vis region of the electromagnetic spectrum.

**Figure 4:**
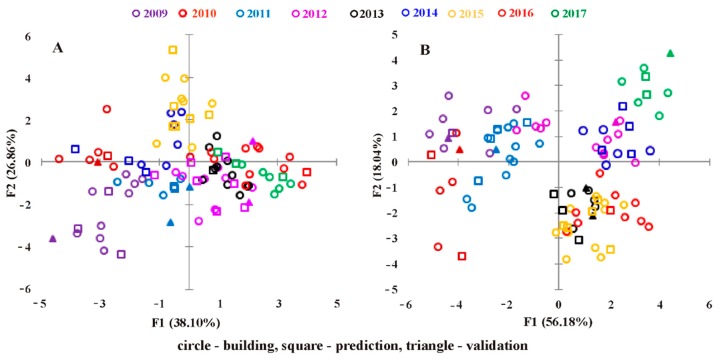
Scatter plot of the first two discriminant functions showing separation of different harvest years: (**A**) UV-Vis data and (**B**) FT-IR data.

For the discrimination of wines according to the harvest year, the LDA models were developed using the 15 PCs resulted by applying the PCA for the experimental data (UV-Vis and FT-IR). Figure 4 shows the score plot of the first two PCs of the LDA model, which contain 64.96% of variance for the UV-Vis data and 74.22% for the FT-IR data from which it can be observed that the grouping is similar to the PCA score plot, with a clear discrimination of aged wines from the 2009, 2010, and 2011 harvest years. In this case, the classification of wine according to the harvest year was better for the LDA based FT-IR data model.

The LDA models had a similar overall rate of correct classification for both, UV-Vis and FT-IR data (67.59% and 62.96%, respectively) (see Supplementary Table S2). Generally, a conclusive result for vintage classification was achieved for all investigated years, less for young wines (2016 and 2017), using UV-Vis data, and for the year 2012 using FT-IR data. The technique of cross-validation applied during the test set validation show that the proposed model appears to be a promising chemometric approach, with classification abilities higher than 70.00% for 2009, 2010, 2012, 2015, and 2017 wines using UV-Vis data and for 2011, 2013, 2014, and 2016 wines using FT-IR data, respectively. Incorrect classification of some wines can be due to the fact that the group centroids for wines produced in some years are too close to each other due to the similarity of the UV-Vis and FT-IR fingerprints.

The results from this study verified that differences exist between the wines from different red wine varieties and harvest years, confirming that the UV-Vis and FT-IR spectra contain important information for discriminating among samples. Although prediction models based on quantitative chromatographic data present better performances for wine varietal and harvest year discriminations [30], the results achieved by using screening UV-Vis and FT-IR spectroscopies should also be encouraged, because these techniques are simple (require minimal sample preparation and no highly skilled personnel for operation), rapid, low-cost, and thus are more accessible for routine investigations. Choosing the appropriate statistical approach for data handling, it is an important aspect for developing applicable authentication methodologies.

Screening methods called also non-target methods based on spectroscopic techniques certainly represent an option accessible to many laboratories interested in the issue of wine authentication. Nevertheless, some key challenges, including guidelines and legislation that regulate both development and validation of non-targeted methodologies, the difficulty of comparing the statistical results obtained with different chemometric software, and the need to develop dedicated software that contains well-defined algorithms, should be clarified.

## 3. Materials and Methods

### 3.1. Samples

Samples of authentic wine produced in a single area (Dobrogea region, Romania) were chosen for this study in an effort to minimize the effects due to the geographical area and winemaking, which could substantially influence the UV-Vis and FT-IR wine fingerprints. Thus, a set of thirty-nine bottles of wine made from different red grape varieties produced at SCDVV Murfatlar covering an aging period of nine years (from 2009 to 2017) were used to build the statistical models (training wine set): Cabernet Sauvignon (n = 8), Merlot (n = 8), Pinot noire (n = 6), Feteasca Neagra (n = 9), and Mamaia (n = 8). For the validation of the proposed statistical models, additional spectral acquisitions were performed, representing 25% of the total acquired spectra.

The wines were produced by microvinification using a classical red wine vinification procedure and kept under similar conditions during and after the winemaking process. The samples were bottled in 750 mL glass bottles and were stored in the cellar before the analysis. A detailed description of the investigated red wine samples and the respective notation used in this study is provided in Supplementary Table S3. 

### 3.2. Spectral Measurements

All samples were equilibrated at a room temperature of 25–30 °C (so that highly repeatable spectral acquisition can be achieved) before spectral measurements and scanned immediately after the wine bottles were opened in order to prevent oxidation reactions. Prior to the UV-Vis and FT-IR measurements, the samples were filtrated through 0.45 µm PTFE membranes in order to remove any possible impurities or turbidity. Further sample preparation was not needed, resulting in a significant reduction in time and costs. For each instrumental technique, three spectra were averaged for samples employed in the calibration step and one spectra for samples included in the validation step. Samples were scanned on a single day to eliminate the instrument drift affecting a particular variety.

UV-Visible Spectroscopy: The UV-Vis spectrophotometric measurements were performed using an SPECORD 250 PLUS spectrophotometer (Analytik Jena, Jena, Germany) equipped with quartz cells with 1 mm path length. Data were collected using the Win Aspect Plus Spectra Manager™ II software (Analytik Jena, Jena, Germany). The absorbance spectra were recorded in the working range 190–800 nm with a step resolution of 1 nm. Deionized water was used for the reference scan.

FT-IR Spectroscopy: All spectra were collected in absorbance mode in the mid infrared (MIR) region (500–4000 cm^−1^) with a resolution of 4 cm^−1^, using an FT-IR spectrometer, Bruker ALPHA-E (Bruker Optik GmbH, Ettlingen, Germany), equipped with an ATR system (Attenuated Total Reflectance) with Eco-ZnSe crystal. The OPUS Spectroscopy Software version 7.0 was used for spectra collection and instrument diagnostics (Bruker Optik GmbH, Ettlingen, Germany). Single beam spectra of the samples were obtained and corrected against the water as background. A total of 32 scans were averaged for each spectrum. The ZnSe crystal was carefully cleaned with ultrapure water between measurements and dried with nitrogen gas after each experiment to ensure the best possible sample spectra. A total of 500 µL of each sample were added directly in the ATR cell sample.

In order to identify the main functional groups that absorb in the UV-Vis and FT-IR regions of the electromagnetic spectrum, a qualitative analysis of the main spectral regions for the investigated wines was performed by comparing with data from the literature.

### 3.3. Multivariate Statistical Data Analysis

In order to classify the wine samples according to variety and harvest year, different pattern recognition techniques, such as principal component analysis (PCA), discriminant partial least squares (PLS-DA), and linear discriminate analysis (LDA), were used as multivariate tools.

Different signal pre-treatment methods such as standard normal variate (SNV) method, smoothing, and second derived Savitzky–Golay derivation transformations can be used in order to improve the statistical model performances and, nevertheless, the obtained results are comparable with those obtained by using raw spectral data [43]. Moreover, numerous studies addressing wine authentication approaches were conducted using raw UV-VIS or FTIR spectral data, without any signal pre-treatment [23,44,46]. In the present study, the spectral data were directly statistically processed, without any prior pretreatment.

Spectral data for the two spectroscopic techniques were processed separately being exported from the specific software in ASCII format for UV-Vis measurements and in DPT format for FT-IR measurements and then imported into the Unscrambler software (version X 10.4; CAMO ASA, Oslo, Norway) for the PCA and PLS-DA modeling, while LDA were calculated using Microsoft Excel 2010 and XLSTAT Addinsoft version 15.5.03.3707 (Addinsoft Inc., New York, U.S.). For each instrumental technique, three replicates of each sample (117 spectra) were used to obtain calibration models, and one measurement for each sample (39 spectra) was used as validation set in the Unscrambler software. Multivariate statistical analysis (PCA, PLS-DA, LDA) were developed using full cross-validation procedure.

PCA transforms a set of correlated response variables into principal components (PCs), generating a new set of non-correlated variables. PCA was used to reduce the dimensionality of the data to a small number of PCs, to visualize the presence of unusual outlier samples, and to examine the possible grouping of samples according to variety and harvest year. Classification techniques: Partial least squares discriminant analysis (PLS-DA) and linear discriminant analysis (LDA) were applied to the pre-selected spectral range from PC. LDA is a supervised classification technique that aims to maximize between-group variance and minimize within-group variance for multivariate data, the number of categories and the samples that belong to each category being previously defined [35]. Discrimination models were developed based on the first 5 PCs, 10 PCs, and 15 PCs scores, seeing as a reduction in size was necessary for all four data matrices, considering the high ratio between the number of original variables and the number of samples. PLS-DA was performed to create a more reasonable regression model.

## 4. Conclusions

This study proves the usefulness of UV-Vis and FT-IR screening techniques coupled with multivariate statistical analysis for red wine varietal classification and vintage year prediction. LDA applied as a classification technique on the four data matrices provided satisfactory classification results, UV-Vis spectroscopy being more appropriate for varietal discrimination of red wines, while FT-IR spectroscopy was more efficient for the prediction of wine vintage year. It was very difficult to discriminate between Cabernet Sauvignon, Merlot, and Pinot noire wines, and thus predicting different blend compositions made from these varieties becomes a challenging topic. Both UV-Vis and FT-IR spectroscopic techniques discriminate wines aged more than six years, due to the formation of new compounds during the wine maturation process. The regression models developed have proven to be good enough to correlate the FT-IR spectral data with wine variety and harvest year. However, the similarities between some wines and the nonselective nature of UV-Vis and FT-IR techniques limit the precision of the classification models.

For a reliable wine authenticity assessment process based on screening spectroscopic techniques, the development of robust spectral databases incorporating as many wine samples (covering the variation related to regional conditions, vineyard management, and winemaking practices) as possible, is encouraged.

## Figures and Tables

**Table 1:** Fingerprint regions (cm^−1^) for stretching and bending vibrations in wines, according to literature reports.

	FT-IR Spectral Regions (cm^−1^)	Groups	Assignment	Reference
**Functional group region 4000–1500 cm^−1^**	3500–3000	–OH	Water, alcohols, and phenols	[39]
	3000–2800	C–H stretching of hydrocarbons –CH3 asymmetric stretching vibration O–H stretching of carboxylic acids	Free phenolic acids and catechins, Polyols (glycerol),	[39]
	2300–2100	C–H combinations vibrations and overtones	Ethanol and sugars	[43]
	1900–1600	O–H stretching C–H3 stretch first overtone C–H2, C–H stretch first overtones	Ethanol, glucose, and water	[43]
	1700	C=O	Organic acids	[39]
	1712–1704	C=O	Esters of hydrolysable tannins, especially derivatives of gallic acid and flavors	[15,22]
	1610–1614 1519–1516	C=C	Aromatic compounds, flavonoids	[15,22]
	1600–1530	C–N	Amino acids and their derivatives	[39]
**Fingerprint region (1500–900 cm^−1^)**	1457–1288	C=O, C=C, –CH2–, C–H, –CH3, O–H	Aldehydes, carboxylic acids, proteins, and esters	[44]
	1250–950	stretching and bending vibrations	Hydrolysable and condensed tannins Glucose, oligo- and polysaccharides, alcohols (ethanol)	[39,45]
	1200 1110–1107 1068–1062	stretching vibration of C–O O–H stretch second overtones	Sugars and organic acids	[15]
	<1000	stretching and bending vibrations	Phosphates, phenolics, mono-substituted phenyl derivatives, unsaturated lipids, carotenoids	[39]

**Table 2:** Statistical parameters of the partial least squares discriminant analysis (PLS-DA) model results in the calibration and validation set for wine varietal and harvest year discrimination based on UV-Vis and FT-IR fingerprinting techniques.

	Fingerprinting Technique: UV-Vis/FT-IR			
Discrimination Criterion	Calibration Set		Validation Set	
	RMSEC	R^2^	RMSEV	R^2^
**Wine Varietal Discrimination**				
**Cabernet Sauvignon**	0.233/0.131	0.668/0.895	0.261/0.169	0.582/0.825
**Feteasca Neagra**	0.175/0.136	0.826/0.859	0.197/0.182	0.780/0.813
**Merlot**	0.202/0.121	0.750/0.911	0.223/0.157	0.694/0.848
**Mamaia**	0.202/0.115	0.750/0.918	0.225/0.151	0.689/0.860
**Pinot Noire**	0.206/0.104	0.673/0.918	0.228/0.135	0.600/0.859
**Harvest Year Discrimination**				
**2009**	0.152/0.115	0.748/0.855	0.174/0.146	0.671/0.769
**2010**	0.168/0.085	0.693/0.922	0.187/0.108	0.620/0.872
**2011**	0.155/0.138	0.748/0.828	0.179/0.177	0.711/0.727
**2012**	0.216/0.129	0.581/0.849	0.243/0.163	0.470/0.762
**2013**	0.204/0.129	0.546/0.816	0.232/0.163	0.414/0.713
**2014**	0.199/0.148	0.569/0.760	0.225/0.184	0.451/0.632
**2015**	0.181/0.114	0.706/0.900	0.204/0.142	0.626/0.844
**2016**	0.167/0.132	0.696/0.754	0.186/0.163	0.624/0.626
**2017**	0.193/0.138	0.597/0.794	0.216/0.173	0.491/0.674

## Supplementary Materials

The following are available online at https://www.mdpi.com/1420-3049/24/22/4166/s1, Figure S1: Raw absorption UV-Vis spectra of red wines: (A) different varieties from the same harvest year and (B) different harvest years of the same wine variety, Figure S2: Typical FTIR spectra of different red wine varieties in the spectroscopic region 3500–900 cm^−1^, Figure S3: Distribution of wine samples with different harvest year on the plane defined by the first 3 PCs: (A) using UV-Vis spectral data and (B) using FT-IR spectral data, Table S1: LDA classification matrix for red wines using (1) 5PCs; (2) 10PCs; (3) 15PCs, Table S2: LDA classification matrix for red wines with different harvest years using 15PCs, Table S3: Investigated red wine samples and the specific notation.
